# Expressions of Grief in Online Discussion Forums—Linguistic Similarities and Differences in Pet and Human Bereavement

**DOI:** 10.1177/0030222820914678

**Published:** 2020-04-04

**Authors:** Minna Lyons, Katie Floyd, Haley McCray, Claire Peddie, Katherine Spurdle, Amelia Tlusty, Charlotte Watkinson, Gayle Brewer

**Affiliations:** School of Psychology, The University of Liverpool, United Kingdom

**Keywords:** bereavement, discussion forums, grief, linguistic analysis, loss, pet

## Abstract

We compared online discussion forum posts related to pet loss to those related to human bereavement. Posts (*N *=* *401) were analyzed using the Linguistic Inquiry and Word Count software for frequencies of word use relevant to bereavement. Words related to anger, sadness, and negative emotions were used at similar frequencies for all grief. Sibling loss was associated with using first person pronouns at higher frequencies, and positive emotion words at lower frequencies than other categories of loss. There were some similarities in partners and pets in the word use related to friends and social connectedness. Words related to religion were highest when writing about losing a child and lowest when losing a pet. Our results highlight the similarities in the vocabulary in pet and human bereavement. Findings demonstrate the importance of online discussion forums for understanding the process of grief and specific relationship types.

Pets represent an important source of comfort and companionship. For owners, they may supplement or even substitute human relationships (Brandes, 2010) and sometimes take a role similar to that of a human child (Owens & Grauerholz, 2018). This form of close pet–owner relationship is particularly common in the modern Western context, characterized by delayed parenthood and less frequent contact with extended families. Evolutionary theory has compared pets to social parasites that gain advantage from human psychological mechanisms facilitating kin altruism (Archer, 1997). If pets are, indeed, regarded as kin-like family members, we would expect psychological distress following the death of a pet to be similar to the distress that follows the loss of an important human relationship. Understanding the related bereavement processes is, therefore, important not only in terms of clinical practice but also for increasing our understanding of the evolutionary enigma of pet keeping (Archer, 1997). In the present study, we examine experiences of pet and human relationship loss through online bereavement discussion forums.

If pet keeping is a by-product of psychological mechanisms evolved for kin altruism, bereavement of a pet should parallel the experience of human death. Previous research indicates that the symptoms of grief (e.g., crying, loneliness, guilt, feeling depressed) are similar irrespective whether the individual is mourning a pet or a human loved one (Wrobel & Dye, 2003). With regard to severity of grief, some have suggested that pet grief is less intense than when grieving for a human (Archer & Winchester, 1994; Eckerd et al., 2016; Rajaram et al., 1993; Stokes et al., 2002; Wrobel & Dye, 2003), whereas others have found the grief intensity to be similar to that of human bereavement (Gerwolls & Labott, 1994). Further, approximately one third of bereaved pet owners experience severe, long-lasting grief that may require professional help (Adams et al., 2000). It appears, therefore, that the death of a pet can have a similar influence on the bereaved as the death of a human.

However, there are characteristics of pet loss and features of the pet–owner relationship that may separate the experience of pet bereavement from the loss of others. First, pet owners often have to make a decision to end their pet’s life, which makes the bereavement less severe than when the pet dies accidentally (Davis et al., 2003; McCutcheon & Fleming, 2002; Stokes et al., 2002). McCutcheon and Fleming (2002) speculated that this is because pet owners who euthanize their pet have more support from the veterinary clinic, which could verify their decision to end the life of the pet. Second, in human bereavement, religion is often used as a tool for trying to find meaning which may result in better coping (Neimeyer, 2015). Although a substantial number of pet owners believe in the afterlife of their pet, religiosity does not appear to relieve their grief (Davis et al., 2003). Finally, grief after pet loss is sometimes described as disenfranchised (i.e., not valued or acknowledged by society as a valid reason for mourning; Bussolari et al., 2018). Disenfranchisement can lead to poor psychological adjustment after bereavement if the grieving person feels that they have limited opportunities to disclose their feelings to others (Juth et al., 2015). This may be especially problematic for people who use pets as a substitute for human relationships (e.g., as a replacement for partners; Adams et al., 2000). Together, these issues suggest that though pets are kin-like, the bereavement process for pet owners may differ from human relationships because of circumstance or societal norms.

Online discussion forums provide a safe place for those who suffer from disenfranchised grief to have their bereavement validated by others (Paulus & Varga, 2015; Varga & Paulus, 2014). Online bereavement support communities unite mourners who share the experience of loss but may not be able to openly discuss their feelings face-to-face with their offline support networks. For example, this may be the case in bereavement where the cause of death is suicide (Bailey et al., 2017; Krysinska & Andriessen, 2013). Online forums allow validation of the grief, psychosocial support, and an opportunity for people to tell their stories (Döveling, 2017; Swartwood et al., 2011). Research has established the importance of virtual communities in supporting those who are bereaved though evidence for the therapeutic benefits of online engagement is sometimes contradictory (Hartig & Viola, 2016; van der Houwen et al., 2010). To our knowledge, previous studies have not yet compared the similarities or differences in the posts relating to human, as opposed to pet bereavement.

In this study, we compare differences in language use in people seeking online peer support after losing a pet, or after losing an important human relationship. Language can provide an important insight into the human experience. For instance, psychological distress has been associated with distinctive linguistic styles such as elevated use of first person personal pronouns (e.g., Edwards & Holtzman, 2017; Lyons et al., 2018; Tackman et al., 2019), possibly due to an increased self-focus and decreased self-distancing. Distress surrounding bereavement also seems to have other characteristic verbal markers, such as using the past tense or anger-related words (Brubaker et al., 2012). In contrast, using language that has a positive tone has been associated with better adjustment after parental loss in children (Wardecker et al., 2017). The present study is the first study to investigate differences in the language used by people who grieve for a pet, as opposed to grieving for a human relationship. Specifically, we compare cat and dog loss language use with loss in relation to different categories of important human relationships (i.e., siblings, partners, and children).

We predict that the frequencies of word use in linguistic categories associated with distress and grief will be similar in those who are grieving for pets (cats or dogs), and those who are grieving a human relationship. However, due to social, practical, and cultural influences on bereavement, we expect that those grieving for a pet will use fewer words related to social processes such as family and friends and personal concerns such as religion, work, or money.

## Method

### Sample and Procedure

We conducted an internet search using terms such as “bereavement discussion forum,” “grief discussion forum,” and “rainbow bridge discussion forum” to source relevant online forum sites. We identified seven suitable discussion forums that were openly available and did not require a registration for viewing user entries. As a well-established popular forum with specific sites for each bereavement category of interest (i.e., pet, child, sibling, spouse), most entries (*n *=* *309) were taken from the https://forums.grieving.com site. After exhausting suitable entries from this site, we gathered the remaining posts from specific pet loss forums (e.g., http://petlossforum.forummotion.com/f3-gen) or other discussion forums that include posts for different categories of loss (e.g., http://www.thelightbeyond.com/forum/). We selected 401 posts in total (100 for spouses, 100 for children, 96 for siblings, 52 for cats, and 53 for dogs). We included posts that (a) were a minimum of 100 words in length, (b) discussed recent deaths (i.e., posts were written within 2 months of the bereavement), (c) were the first post within a thread (i.e., not subsequent posts, nor the responses), and (d) focused on a single death (rather than multiple simultaneous deaths).

### Data Analysis

We used the Linguistic Inquiry and Word Count program (Pennebaker et al., 2015) to analyze the discussion forum entries. The program uses a psychometrically validated dictionary of more than 6,400 words, word stems, and emoticons classified under specific categories such as linguistic dimensions (pronouns, adverbs, articles) and psychological processes (e.g., affective processes, social processes, personal concerns). In the present study, we analyzed the linguistic dimensions of personal pronouns, psychological dimensions of affective words, social processes, time orientation, and personal concerns.

The personal pronouns analyzed in this study were (a) first person singular (e.g., I, me), (b) first person plural (e.g., us, we), (c) second person (e.g., you, yours), (d) third person singular (e.g., he, his), and (e) third person plural (e.g., they, theirs). For the affective processes, we analyzed (a) positive emotion (e.g., love, nice), (b) negative emotion (e.g., hurt, nasty), (c) anger (e.g., hate, annoyed), (d) anxiety (e.g., worried, fearful), and (e) sadness (e.g., crying, sad).

For the social processes, we analyzed words related to (a) family (e.g., daughter, aunt) and (b) friends (e.g., buddy, neighbor). For the time orientation, we analyzed (a) the past (e.g., ago, talked), (b) present (e.g., today, now), and (c) future (e.g., will, soon). For the personal concerns, we analyzed words related to (a) work (e.g., job, boss), (b) leisure (e.g., cook, chat), (c) home (e.g., kitchen, landlord), (d) money (e.g., cash, owe), and (e) religion (e.g., altar, God). This resulted in 18 analyses of variance (ANOVAs); therefore, in order to avoid Type 1 errors, we lowered the accepted alpha for significance to *p *<* *.02.

## Results

To analyze language use differences in people experiencing distinct categories of bereavement, we conducted 18 one-way ANOVAs. The grief category (i.e., cat, dog, child, sibling, partner) was the independent variable, and the 18 language use categories were the dependent variables. Due to multiple testing of the same data, we lowered the accepted alpha value to *p* < .02. We report post hoc analyses (including interaction plots) only for those ANOVAs that reached this significance level. Tables 1 [Table table2-0030222820914678][Table table3-0030222820914678]to 4 also present the means and standard deviations for all dependent variables.

**Table 1.** LIWC Analysis for the Use of Personal Pronouns.

	Cat	Dog	Child	Sibling	Partner	Main effect		
	*M* (*SD*)	*M* (*SD*)	*M* (*SD*)	*M* (*SD*)	*M* (*SD*)	*F*	*p*
FPS	8.42 (2.87)	7.85 (2.84)	8.39 (3.43)	9.51 (2.97)	8.89 (2.60)	3.36	.01
FPP	1.11 (1.52)	1.97 (2.14)	1.27 (1.32)	0.92 (0.97)	1.79 (1.07)	8.24	.001
SPS	0.66 (1.99)	0.40 (1.28)	0.36 (0.61)	0.38 (1.07)	0.22 (0.49)	1.37	.24
TPS	5.94 (2.61)	6.09 (2.27)	5.15 (2.28)	5.36 (2.53)	5.30 (2.50)	1.95	.10
TPP	0.31 (0.32)	0.48 (0.73)	0.54 (0.70)	0.40 (0.51)	0.44 (0.58)	4.60	.18

*Note.* LIWC = Linguistic Inquiry and Word Count; FPS = first person singular; FPP = first person plural; SPS = second person singular; TPS = third person singular; TPP = third person plural; M = mean; SD = standard deviation.

**Table 2. table2-0030222820914678:** LIWC Analysis for the Use of Affective and Social Words.

	Cat	Dog	Child	Sibling	Partner	Main effect	
	*M* (*SD*)	*M* (*SD*)	*M* (*SD*)	*M* (*SD*)	*M* (*SD*)	*F*	*p*
POS	2.87 (1.21)	2.91 (1.20)	2.93 (1.47)	2.31 (1.23)	2.64 (1.32)	3.40	.01
NEG	3.44 (1.43)	3.53 (1.55)	3.06 (1.78)	3.57 (1.60)	3.19 (1.26)	2.82	.13
ANX	0.53 (0.55)	0.48 (0.52)	0.37 (0.48)	0.55 (0.55)	0.48 (0.46)	1.74	.14
ANG	0.40 (0.47)	0.37 (0.50)	0.45 (0.50)	0.58 (0.61)	0.50 (0.60)	1.71	.15
SAD	1.78 (1.06)	1.81 (1.46)	1.44 (1.10)	1.54 (1.16)	1.43 (0.89)	1.70	.15
FAM	0.63 (0.75)	0.89 (0.70)	2.10 (1.25)	2.70 (1.42)	1.29 (1.06)	42.95	.001
FRI.	0.58 (0.71)	0.41 (0.46)	0.31 (0.43)	0.35 (0.41)	0.58 (0.55)	5.42	.001

*Note.* LIWC = Linguistic Inquiry and Word Count; POS = positive emotion; NEG = negative emotion; ANX = anxiety; ANG = anger; SAD = sadness; FAM = family; FRI = friend; M = mean; SD = standard deviation.

**Table 3. table3-0030222820914678:** LIWC Analysis for Use of Personal Concern Words.

	Cat	Dog	Child	Sibling	Partner	Main effect	
	*M* (*SD*)	*M* (*SD*)	*M* (*SD*)	*M* (*SD*)	*M* (*SD*)	*F*	*p*
WOR	0.63 (0.49)	0.59 (0.52)	1.03 (1.04)	0.87 (0.82)	0.87 (0.67)	3.78	.005
LEI	0.43 (0.40)	0.59 (0.69)	0.57 (0.61)	0.62 (0.60)	0.60 (0.54)	1.04	.380
HOM	1.09 (0.72)	0.88 (0.64)	0.57 (0.61)	0.59 (0.60)	0.84 (0.77)	5.71	.001
MON	0.15 (0.27)	0.16 (0.33)	0.58 (1.16)	0.23 (0.42)	0.28 (0.42)	3.09	.02
REL	0.20 (0.37)	0.16 (0.33)	0.58 (1.18)	0.23 (0.42)	0.28 (0.45)	5.32	.001

*Note.* LIWC = Linguistic Inquiry and Word Count; WOR = work; LEI = leisure; HOM = home; MON = money; REL = religion; M = mean; SD = standard deviation.

**Table 4.** LIWC Analysis for the Use of Time Orientation Words.

	Cat	Dog	Child	Sibling	Partner	Main effect	
	*M* (*SD*)	*M* (*SD*)	*M* (*SD*)	*M* (*SD*)	*M* (*SD*)	*F*	*p*
PAS	8.13 (2.17)	8.51 (2.79)	7.21 (2.55)	7.82 (2.48)	8.56 (2.60)	4.24	.004
PRE	9.07 (2.83)	9.44 (3.33)	10.70 (3.74)	10.62 (3.36)	10.17 (3.61)	2.91	.02
FUT	0.97 (0.85)	0.87 (0.61)	1.33 (0.87)	1.01 (0.65)	1.33 (10.75)	6.32	.001

*Note.* LIWC = Linguistic Inquiry and Word Count; PAS = past; PRE = present; FUT = future; M = mean; SD = standard deviation.

**Figure 1.**
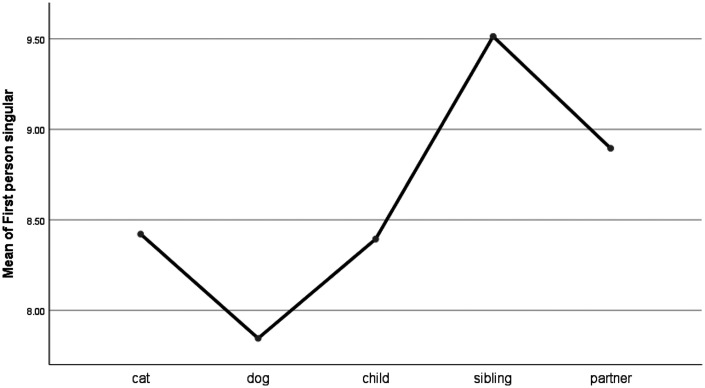
Means Plot for the Use of First Person Singular Words.

**Figure 2.**
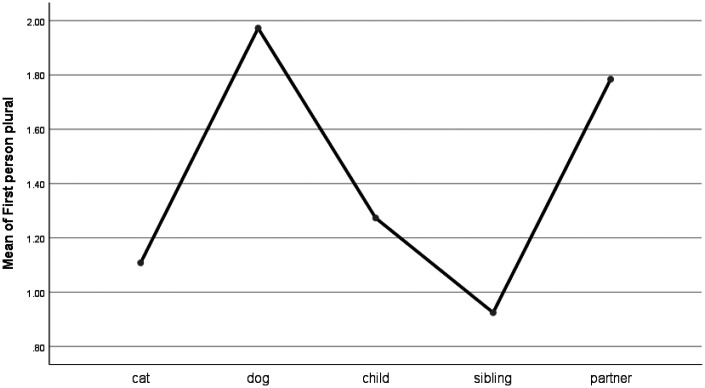
Means Plot for the Use of First Person Plural Words.

For personal pronoun use, only the first person singular (e.g., I, me, my) and plural (e.g., we, us, ours) emerged as significantly different between the grief categories (see Table 1 and Figures 1 and 2). The post hoc analyses (Least Significant Difference) indicated that people used first person singulars significantly more frequently when they talk about their siblings than they do when they talk about their pets. However, there were no significant differences in the use of first person singulars when people talked about their pets and their children or partners. People who grieved for their dogs and partners used the first person plural (e.g., us, we, our) more than people who grieved for their cats, children, or siblings.

**Figure 3.**
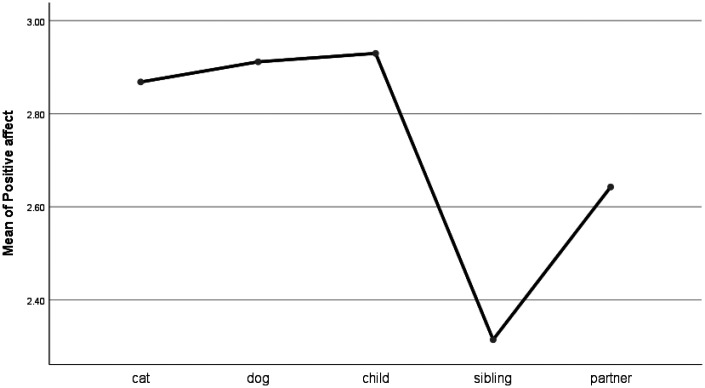
Means Plot for Use of Positive Affect Words.

For affective words, significant group differences were found for positive words (e.g., love, nice; see Table 2 and Figure 3). Posts relating to cats and dogs were significantly different from sibling posts only. When talking about deceased pets, people used positive words at a similar frequency, as they do when talking about children and partners. People who were writing posts about sibling bereavement used significantly fewer positive words than the other groups.

**Figure 4.**
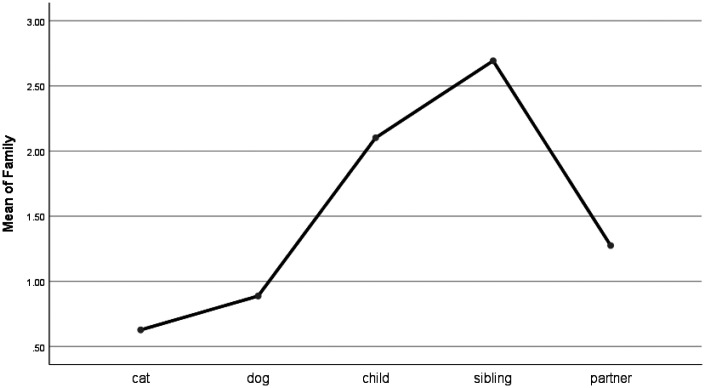
Means Plot for Use of Family Words.

**Figure 5.**
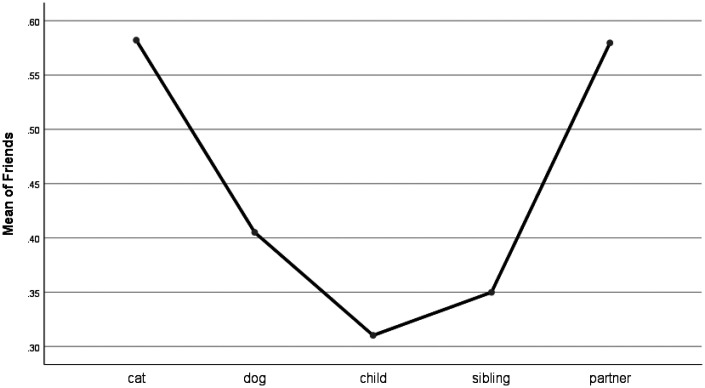
Means Plot for Use of Friend Words.

For words describing social processes, there were significant group differences for the use of family words (e.g., daughter, aunt). People who talked about their pets used significantly fewer family words than people who talked about all categories of human bereavement (see Figure 4). For words describing friendship, there were significant differences between cats, and children and siblings. Friend words (e.g., buddy, neighbor) were used more frequently for cats than for children and siblings (see Figure 5). Indeed, the highest frequencies of friend words were used for cats and partners.

**Figure 6.**
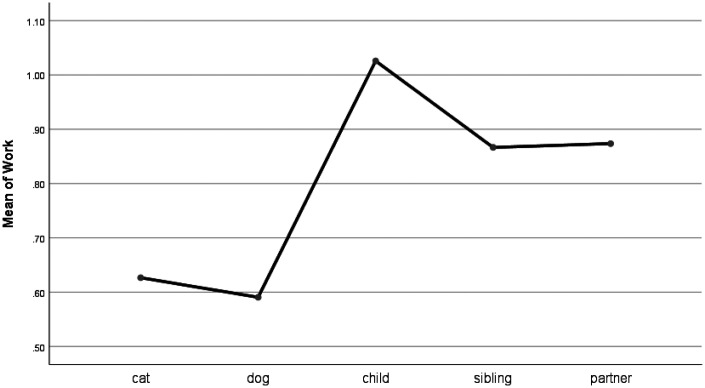
Means Plot for Use of Work Words.

**Figure 7.**
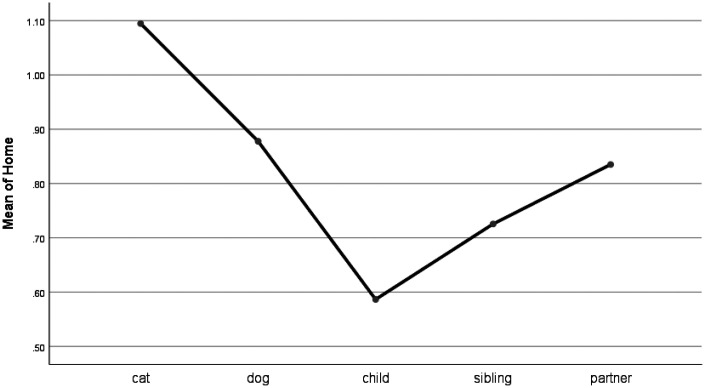
Means Plot for Use of Home Words.

**Figure 8.**
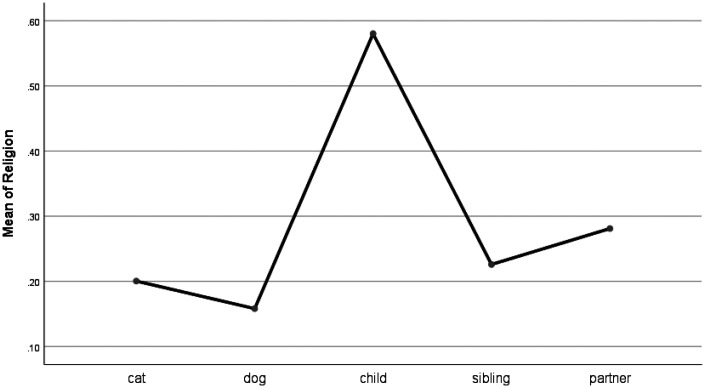
Means Plot for Use of Religion Words.

For words describing work (e.g., job, boss), pet forum posts were similar to other groups, though work-related words were used at a significantly lower frequency for pets than when talking about a bereavement of a child (see Figure 6). Home words (e.g., kitchen, landlord) were used at a significantly higher frequency when talking about cats and dogs than when talking about children. Also, people used more home words when writing about cats than they did when posting about siblings (see Figure 7). Religion-related words (e.g., altar, God) were used at higher frequency for children than for pets and at similar frequency for pets and siblings and partners (see Figure 8).

**Figure 9.**
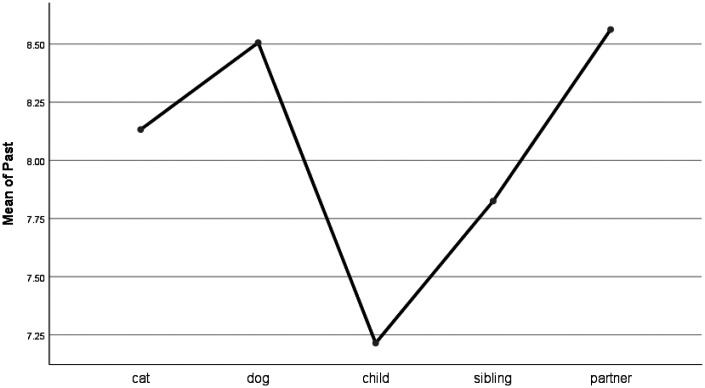
Means Plot for Use of Past Words.

**Figure 10.**
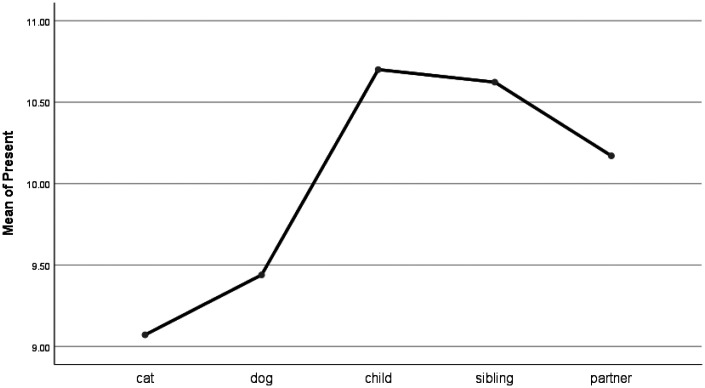
Means Plot for Use of Present Words.

**Figure 11.**
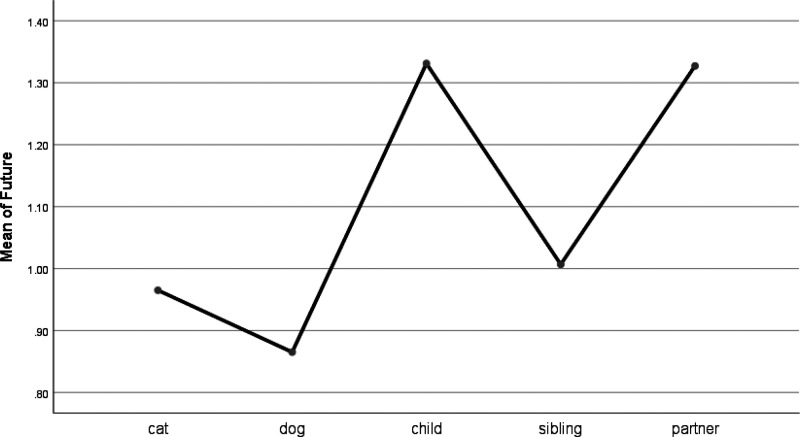
Means Plot for Use of Future Words.

People were less likely to adopt the past tense (e.g., ago, talked) when talking about children than when talking about pets (see Table 4 and Figure 9). There were no significant differences between pets and partners or siblings when writing in the past tense. People used the present tense (e.g., today, now) much less when talking about their pets than when talking about their children or siblings (see Figure 10). Further, people used significantly fewer future-oriented words (e.g., will, soon) when talking about their pets than they did when writing about their partners or children (see Figure 11).

## Discussion

The key finding of the present study was the overall similarity of language use when people wrote about loss of their pets and when they wrote about loss of significant human relationships. Words related to affective processes (e.g., sadness, anger, anxiety) that are relevant in grieving were used at the same frequencies irrespective of the type of grief. First person singular use was similar in pet grief posts as in posts about partners or children. First person singular use has been related to mental distress (Edwards & Holtzman, 2017; Lyons et al., 2018), potentially reflecting increased self-focus and rumination. Our findings suggest that indeed, pet grief is similar to grieving for humans and should be taken seriously in clinical practice (Chur-Hansen, 2010) and grief counselling (Cordaro, 2012).

Interestingly, sibling bereavement seemed to be different to other types of grief. Online discussion forums for sibling bereavement contained fewer positive words, and more first person singular pronouns than other discussion forums. Sibling relations are characterized by both conflict and closeness, which may increase the complexity of emotions during bereavement and severity of grief (Smigelsky et al., 2019). Relationships with pets can be less conflict-ridden and more satisfactory than those that people have with their sisters and brothers (Cassels et al., 2017). Thus, the grieving process might be less complicated when losing a pet than when losing a sibling, which could be reflected in fewer first person singular, and more positive emotion words in pet loss.

There were some interesting similarities between pet and partner-oriented language. For instance, online discussion forum posts used the first person plural (e.g., we, us) more when talking about partners and dogs, than cats, siblings, and children. Increased “we” talk indicates better relationship functioning, social connectedness, and a shared group identity (Tausczik & Pennebaker, 2010), as well as healthier attachment with a romantic partner (Dunlop et al., 2019). In a bereavement context, greater use of words indicating social connectedness has been related to better coping in children (Kaplow et al., 2018; Wood et al., 2005). Dogs have a unique role in the lives of their owners in increasing social connectedness (Kotrschal, 2018), which could be echoed in the style of the owner’s post-mortem language use. Interestingly, both dogs and spouses (relative to other types of relationship partners) are important attachment figures that people turn to during difficult times (Beetz et al., 2012; Kurdek, 2009). The special connection with romantic partners and dogs could be reflected in increased adaptive word use when sharing feelings surrounding bereavement.

Friend words were used most frequently for posts involving cats and partners, and there were significant differences between the frequency of friend words used for posts related to cats, and children and siblings. This is interesting taking the extensive research focus on establishing dogs as “man’s best friends” (MacKay et al., 2016) and a dearth of data on cat-owner bonds (Vitale & Udell, 2019). It is clear that the attachment to pet cats is important (Stammbach & Turner, 1999) and can parallel that of attachment to pet dogs and partners in the comfort and companionship that they provide. Another possibility for the increased use of friend words in these bereavement categories is that bereaved people draw more on social support from their friends when battling with grief relating to the loss of a cat or a partner, and the posts included mentions of these friends. Either way, our results are intriguing, and call for more research on cat–human bonds in pet-owning families.

Another significant finding was that people who talked about their pets used significantly fewer family words than people who talked about human bereavement in all categories. Again, this could either be because pets are referred to less in terms of being family members or because there is a lack of understanding from the family who may view pet bereavement as abnormal (Williams & Green, 2016). The latter option perhaps is more likely, as bereaved pet owners are less likely to receive familial support than when grieving for another person and pets are, indeed, often viewed as family members by the bereaved (McNicholas et al., 2005).

For personal concern words such as religion, pet posts were similar to sibling and partner posts, all of which differed from children-oriented posts. Words relating to religion were used more frequently by those grieving for a child than other bereavement groups. The loss of a child is a devastating and somewhat unnatural life event, as parents would normally expect to die before their offspring. Perhaps, one way of coping is to turn to religion to appraise the death through religious beliefs and allow the continuation of relationship with the deceased via beliefs in afterlife (Benore & Park, 2004; Sanders, 1980). This seems to be most important for those who had lost a child and least important for those who had lost a pet.

Online discussion forum posts involving pets were less likely to adopt the present tense than those discussing children or siblings. Similarly, forum posts describing the loss of a pet were less likely to use future words than when discussing a partner or child. Those grieving for a partner or child may mourn events that they will never experience (e.g., retirement with a partner or a child graduating; Rubin, 1990). Those grieving for a partner may also face an uncertain future (e.g., loss of income and sole responsibility for their children). Hence, the greater emphasis on current or future events in these groups may reflect both practical and psychosocial issues. The focus on lost current or future events may, in part, also explain less frequent use of the past tense when writing about children compared with pets.

The data collected through the method of open access discussion forums are not without their limitations. First, we could not control for different types of death. There could be differences in bereavement and language use depending on how the individual died. For example, in human bereavement, people use more anger and sadness words when writing about suicide victims than about people who died of natural causes (Lester, 2012; although see also Scourfield et al., 2019 for a study that found no differences between road accidents and suicides). Also, severity of pet bereavement seems to depend on whether the owner made a decision to euthanize their pet (Davis et al., 2003; McCutcheon & Fleming, 2002; Stokes et al., 2002). As this information could not be collected for all posts, this could not be analyzed. Similarly, responses to loss of a pet may vary cross-culturally (Bussolari et al., 2017), and while all online discussion forum posts were written in English, it was not possible to determine the country of origin. However, these are factors that influence bereavement and the language use around it and should be investigated in future studies with larger samples of posts.

To conclude, we compared online discussion forum posts written after losing a pet to those written after losing a sibling, child, or partner. The language used by those mourning the loss of a pet demonstrated important similarities to the text written by those experiencing other forms of bereavement. For example, words related to grief, anger, sadness, and negative emotions were used with similar frequencies by those grieving for pets and human relationships. These findings are consistent with previous research highlighting commonalities of pet and human bereavement and suggest that the loss of a pet receive greater attention. Findings also demonstrate the importance of online discussion forums for understanding the process of grief and specific relationship types.
